# Supplementary material for: Can resting heart rate explain the heart rate and parasympathetic responses during rest, exercise, and recovery?
Source: PLoS One. 2022 Dec 7;17(12):e0277848. doi: 10.1371/journal.pone.0277848 (PMC9728889; doi:10.1371/journal.pone.0277848)
Supplement: Attachment — Submitted filename: comments.docx [file pone.0277848.s002.docx]

In the present investigation the authors sought to investigate the relationship between resting, exercise and recovery HR and HRV (only the time-domain HRV index RMSSD). They divided the participants into two groups according to their resting HR (Bradycardic vs Normocardic Group). The manuscript needs extensive revision before being considered for publication as some statements and considerations are not totally convincing. The manuscript also needs revision for language and grammar. Please consider the following suggestions.

Abstract:

L14: you may prefer using dynamics instead of dynamic

L14: please change into “remains to be clarified”

L17: what do you mean with patterns? That sounds like just the average value of HR at rest in the supine position..am I correct?

L23: please mention that this “manoeuvre” (i.e. the change in posture) is part of your evaluation protocol. Indeed, it seems that you wonder whether individuals with a low resting HR in the supine position have lower values of HR in the orthostatic position and/or different responses to the change in posture than individuals with a higher resting HR

Introduction:

L40: please clarify “chronotropic reserve”, that is usually defined considering both resting HR and HRmax

L43: and what happens to HR? this seems a good spot to introduce HRR

L44: what do you mean? A higher parasympathetic activity at rest would reflect into a faster parasympathetic recovery after exercise and/or to a faster para response to an active manoeuvres, such as an orthostatic challenge? I would delete this sentence..at this point of the introduction is still not clear how this two phenomena would be related

L47: consider diving this long sentence into shorter sentences

L54: you probably forgot “activity”; in my opinion HRV could be introduced earlier in the manuscript as the assessment of parasympathetic activity in the study you mention mainly relies on that

L58: and what did they found? what kind of HR (sub-max or max)? It is not clear how resting HR is related to exercising HR. Is it that individuals with a low HR tend to have low values of HR during exercise for a given sub-max exercise intensity?

L63: please provide some references to your statements

L74: what are these RHR profiles? You are using RHR as an abbreviation for resting heart rate, but here you are probably intending that is possible to characterize/classify an individual’s cardiac autonomic profile via measurements of HR and HRV indices. If I am not wrong, you want to test the hypothesis that resting, exercise and recovery HR and HRV responses can be somewhat related and that information obtained from HR and HRV evaluations at rest can inform about an individual’s ability to responds to stressful tasks such as exercise. Please consider rephrasing these last sentences.

L81: what is your hypothesis?

L83: please consider not using patterns if RHR is just the average value of HR (bpm) without any other information about its variability

Methods

L103: you might not need “RHR” here

L128: which threshold did you use?

L137: what is the physiological relevance of HR initial?

L137: maximum “predicted” HR.

L151: over a 5-min time window?

L155: over a 1-min time window? From end EX– 1 min to end EX ?

L158: please move this paragraph before the description of the analysis you conducted on HR/HRV indices, otherwise it is hard to follow

L158: this is a very important part for the study outcomes being HRR/HRV recovery influenced by the exercise intensity domain. Please clarify that 85% is the target to be reached in terms of predicted HRmax. Is there any other measurement that can inform about exercise intensity from a metabolic point of view? VO_2_?

L164: were participants allowed to run? The average duration of the test is 7.5 min, according to the protocol at 7.5 min the exercise intensity is 9 km/h. Was the 2 initial min enough to reach a steady value of HR before further increasing the treadmill velocity?

L195: trivial effect instead of no effect

Results

Please state in the tables whether the values refer to mean or median values

Discussion

L253: still not clear what RHR profiles means

L257: how? The two groups have the same average age..so the predicted HRmax is on average the same, the difference in resting HR directly reflects into a different “estimated” chronotropic reserve

What are the potential issues you may face targeting 85% of the predicted HR max in two groups that differ in their resting HR? are there any? Consider discussing this in the limitations section

L257: how did you measure para withdrawal during exercise? That is not clear at all. Is there a difference in HR kinetics? Is there a faster HR response in BG? The difference in reactivity refers to the orthostatic task.

L258: HRR 1^st^ min should reflect parasympathetic reactivation but this is not different in the two groups. Please elaborate on this. HRR 5^th^ min should reflect para reactivation + sympathetic withdrawal occurring during the post-exercise period, but you are not properly discussing this in the manuscript. HRR30s is also often used as a marker of parasympathetic reactivation. Why did you choose not to present those data?

L265: this part is really similar to the first part of the introduction. Please consider rephrasing

L271-L283: this part is not very comprehensible. Please try to make your point more clear rearranging these three sentences and avoiding repetitions

L288: for a similar resting HR can HRV analysis add some meaningful information to interpret subject’s cardiac autonomic profile and responses to stressful tasks?

L351: so why have you found the differences 3 and 5 min post exercise? How does resting HR can influence your calculations? If you try to consider resting HR do these differences disappear?

BG starts from 82 bpm and NG from 88 bpm, they both go up to 164 then down to 97 bpm and 104 bpm 5 min post-exercise. So after 5 min both groups are around + 15 bpm over their initial HR, and the difference seems to be similar to the difference at rest. If you consider the higher starting point for NG group the difference becomes further smaller. So, are they different? Or not?

This part is really important for the general purpose of the manuscript. It needs to be better presented and discussed.

What about adding some graphs?

How much of the variance in post-exercise HRR (1^st^, 3^rd^, 5^th^ min of rec) or in reactivity to an orthostatic test can be explained by the differences in term of resting HR? what percentage of explained variance is enough to safely say that measuring HR/HRV at rest is helpful instead of including other type of measurements? Please clarify.
