# Supplementary material for: Can resting heart rate explain the heart rate and parasympathetic responses during rest, exercise, and recovery?
Source: PLoS One. 2022 Dec 7;17(12):e0277848. doi: 10.1371/journal.pone.0277848 (PMC9728889; doi:10.1371/journal.pone.0277848)
Supplement: Attachment — Submitted filename: responses_to_reviewers_plos.docx [file pone.0277848.s003.docx]

Responses to the reviewers

2022/08/26

Manuscript title: Can resting heart rate explain the heart rate and parasympathetic responses during rest, exercise, and recovery?

Manuscript ID: PONE-D-22-17046.

Authors: Giliard Lago Garcia, Luiz Guilherme Grossi Porto, Carlos Janssen Gomes da Cruz and Guilherme Eckhardt Molina

We thank the Editor-in-Chief of PLOS ONE for the opportunity to resubmit our paper, and we are very grateful to the reviewers for their comments and suggestions, which importantly improved our manuscript.

As requested, we have highlighted edits to facilitate further review. We believe that the responses and additions satisfactorily address the reviewers' comments and suggestions and hope you will agree. The edits have clarified several points and have resulted in an improved manuscript, which we hope is now ready for final acceptance.

**# To Journal Requirements:**

When submitting your revision, we need you to address these additional requirements.

1. Please ensure that your manuscript meets PLOS ONE's style requirements, including those for file naming. The PLOS ONE style templates can be found at [https://journals.plos.org/plosone/s/file?id=wjVg/PLOSOne_formatting_sample_main_body.pdf](about:blank) and [https://journals.plos.org/plosone/s/file?id=ba62/PLOSOne_formatting_sample_title_authors_affiliations.pdf](about:blank)

We adjusted as requested.

2. Please include your tables as part of your main manuscript and remove the individual files. Please note that supplementary tables (should remain/ be uploaded) as separate "supporting information" files.

We adjusted as requested.

3. Thank you for stating the following financial disclosure:

"Yes. This work was supported by Fundação De Apoio À Pesquisa Do Distrito Federal - FAPDF under Grant 11/2022"

Please state what role the funders took in the study.  If the funders had no role, please state: "The funders had no role in study design, data collection and analysis, decision to publish, or preparation of the manuscript." 
If this statement is not correct you must amend it as needed. 
Please include this amended Role of Funder statement in your cover letter; we will change the online submission form on your behalf.

We adjusted as requested.

4. Thank you for stating the following in the Funding Section of your manuscript:

"This work was supported by Fundação De Apoio À Pesquisa Do Distrito Federal - FAPDF under Grant 11/2022."

We note that you have provided funding information that is not currently declared in your Funding Statement. However, funding information should not appear in the Acknowledgments section or other areas of your manuscript. We will only publish funding information present in the Funding Statement section of the online submission form. 
Please remove any funding-related text from the manuscript and let us know how you would like to update your Funding Statement. Currently, your Funding Statement reads as follows:

"Yes. This work was supported by Fundação De Apoio À Pesquisa Do Distrito Federal - FAPDF under Grant 11/2022"

Please include your amended statements within your cover letter; we will change the online submission form on your behalf.

We adjusted as requested.

5. In your Data Availability statement, you have not specified where the minimal data set underlying the results described in your manuscript can be found. PLOS defines a study's minimal data set as the underlying data used to reach the conclusions drawn in the manuscript and any additional data required to replicate the reported study findings in their entirety. All PLOS journals require that the minimal data set be made fully available. For more information about our data policy, please see [http://journals.plos.org/plosone/s/data-availability](about:blank).

Upon re-submitting your revised manuscript, please upload your study's minimal underlying data set as either Supporting Information files or to a stable, public repository and include the relevant URLs, DOIs, or accession numbers within your revised cover letter. For a list of acceptable repositories, please see [http://journals.plos.org/plosone/s/data-availability#loc-recommended-repositories](about:blank#loc-recommended-repositories). Any potentially identifying patient information must be fully anonymized.

Important: If there are ethical or legal restrictions to sharing your data publicly, please explain these restrictions in detail. Please see our guidelines for more information on what we consider unacceptable restrictions to publicly sharing data: [http://journals.plos.org/plosone/s/data-availability#loc-unacceptable-data-access-restrictions](about:blank#loc-unacceptable-data-access-restrictions). Note that it is not acceptable for the authors to be the sole named individuals responsible for ensuring data access.

We will update your Data Availability statement to reflect the information you provide in your cover letter.

We adjusted as requested.

**# To the Reviewer 1**

**Abstract:**

L14: you may prefer using dynamics instead of dynamic

We adjusted as requested.

**# New word in the Abstract, page 2, line 25 (highlighted)**

*… dynamics…*

L14: please change into "remains to be clarified"

**# New word in the Abstract, page 2, line 26 (highlighted)**

*… remain to be clarified …*

L17: what do you mean with patterns? That sounds like just the average value of HR at rest in the supine position.am I correct?

Thank you very much for the question. You are correct in your understanding, which was adjusted in the abstract.

**# New words in the Abstract, page 2, line 28 (highlighted)**

*… RHR average values …*

L23: please mention that this "manoeuvre" (i.e. the change in posture) is part of your evaluation protocol. Indeed, it seems that you wonder whether individuals with a low resting HR in the supine position have lower values of HR in the orthostatic position and/or different responses to the change in posture than individuals with a higher resting HR

We adjusted as requested.

**# New phase in the Abstract, page 2, lines 31 - 34 (highlighted)**

*A Polar RS800® was used to record the RR-interval series and HR at rest in the supine position, following the postural change (from supine to orthostatic position) and in the orthostatic position for 5 minutes, as well as during and after a submaximal exercise testing.*

**Introduction:**

L40: please clarify "chronotropic reserve", that is usually defined considering both resting HR and HRmax

Thank you very much for the question.

**# Removed words in the Introduction, pages 2, line 55**

*… (chronotropic reserve).*

**# New phrase in the Introduction, pages 3, lines 69 – 72 (highlighted)**

*When evaluating the HR during the exercise, only one study showed a significantly positive association between parasympathetic activity at rest with chronotropic reserve (CR); the difference between HR at peak exercise and the RHR in the supine position, during the maximal exercise test [16].*

In the section Material and Methods on page 07 and lines166 - 167, we defined chronotropic reserve as the difference between HR_peak_ from RHR, according to Jouven 2005 and Lauer, 1996.

JOUVEN, Xavier, et al. "Heart-rate profile during exercise as a predictor of sudden death." New England journal of medicine 352.19 (2005): 1951-1958.

LAUER, Michael S., et al. "Impaired heart rate response to graded exercise: prognostic implications of chronotropic incompetence in the Framingham Heart Study." Circulation 93.8 (1996): 1520-1526.

L43: and what happens to HR? this seems a good spot to introduce HRR

Thank you very much for their comments. Consequently, we have adjusted as suggested.

**# New phase in the Introduction, page 2, lines 56 - 58 (highlighted)**

*In contrast, after exercise, the short-term post-exercise HR adaptation, the heart rate recovery (HRR), occurs in response to simultaneous rapid parasympathetic reactivation and progressive sympathetic deactivation [5].*

L44: what do you mean? A higher parasympathetic activity at rest would reflect into a faster parasympathetic recovery after exercise and/or to a faster para response to an active manoeuvres, such as an orthostatic challenge? I would delete this sentence..at this point of the introduction is still not clear how this two phenomena would be related

We adjusted as requested by the Reviewer.

**# Removed phase in the Introduction, pages 3, line 60**

*… Therefore, it is reasonable to expect that these chronotropic and autonomic responses to exercise-induced stress are dependent on the resting cardiac autonomic condition.*

L47: consider diving this long sentence into shorter sentences

We adjusted as requested, and we rewrote this paragraph.

**# New paragraph in the Introduction, page 3, lines 59 - 61 (highlighted)**

*Consequently, a high RHR, slow HR response during an exercise test, and a delayed HRR are associated with reduced parasympathetic activity, withdrawal impairment, and reactivation of parasympathetic activity, respectively [1-3, 6, 7].*

L54: you probably forgot "activity"; in my opinion HRV could be introduced earlier in the manuscript as the assessment of parasympathetic activity in the study you mention mainly relies on that

We adjusted as requested. We did not introduce earlier HRV in the manuscript because we presented the cardiac autonomic modulation phenomenon that regulates heart rate at rest, during exercise, and during recovery in the previous paragraph.

**# New word in the Introduction, pages 3, line 64**

*… parasympathetic activity at rest...*

L58: and what did they found? what kind of HR (sub-max or max)? It is not clear how resting HR is related to exercising HR. Is it that individuals with a low HR tend to have low values of HR during exercise for a given sub-max exercise intensity?

We adjusted as requested, and we rewrote the paragraph.

**# New paragraph in the Introduction, pages 3, lines 67 – 75 (highlighted)**

*In this clinical setting, some studies have reported a significant association between resting parasympathetic activity with HRR after a maximal or submaximal incremental exercise test [9, 11, 14-16]. When evaluating the HR during the exercise, only one study showed a significantly positive association between parasympathetic activity at rest with chronotropic reserve (CR); the difference between HR at peak exercise and the RHR in the supine position, during the maximal exercise test [16]. On the other hand, other studies have not shown a significant association between parasympathetic activity at rest with HRR or parasympathetic activity after incremental exercise tests [8, 10, 12, 13].*

L63: please provide some references to your statements

We adjusted as requested

**# Two citations were inserted in the Introduction, page 3, line 80 (highlighted)**

[5] Michael, Scott; GRAHAM, Kenneth S.; DAVIS, Glen M. Cardiac autonomic responses during exercise and post-exercise recovery using heart rate variability and systolic time intervals—a review. Frontiers in physiology, v. 8, p. 301, 2017.

[17] Garcia, Giliard Lago et al. Effect of different recovery protocols on the cardiac autonomic function. Revista Brasileira de Medicina do Esporte, v. 23, p. 16-20, 2017.

L74: what are these RHR profiles? You are using RHR as an abbreviation for resting heart rate, but here you are probably intending that is possible to characterize/classify an individual's cardiac autonomic profile via measurements of HR and HRV indices. If I am not wrong, you want to test the hypothesis that resting, exercise and recovery HR and HRV responses can be somewhat related and that information obtained from HR and HRV evaluations at rest can inform about an individual's ability to responds to stressful tasks such as exercise. Please consider rephrasing these last sentences.

Thank you very much for the question, and we agree with your statement. The text was adjusted.

**# New phrase in the Introduction, pages 4, lines 85, 87, 89 and 99**

*… RHR average values …*

**# New phrase in the Introduction, pages 4, line 90**

*… preliminary tool for decision-making (i.e., stress management)…*

L81: what is your hypothesis?

**# New phrase in the Introduction, pages 4, lines 94 - 96**

*Therefore, we hypothesize that physically active young men with different RHR average values in the supine position show different HR and parasympathetic activity responses at rest, during, and after a submaximal exercise test.*

L83: please consider not using patterns if RHR is just the average value of HR (bpm) without any other information about its variability

We adjusted as requested.

**Methods**

L103: you might not need "RHR" here

We adjusted as requested.

L128: which threshold did you use?

We adjusted as requested.

**# New phrase in the Material and Methods, page 6, lines 149 - 152**

*We used the medium threshold method that only removed the visually observed ectopic points, as long as the tracing did not lose the physiological pattern and the removal did not exceed 1% of the recording. [19, 20].*

L137: what is the physiological relevance of HR initial?

We thank the Reviewer's question. The physiological relevance of HR initial is related to the central neural control, termed central command, which operates in a feedforward manner changing heart rate response in distinctive degrees at the beginning of the exercise. Therefore, we included this variable in our study protocol to consider its relevance (neural response in the heart) prior to the exercise.

L137: maximum "predicted" HR.

We adjusted as requested.

**# New word in the Material and Methods, page 7, line 167**

*… predicted …*

L151: over a 5-min time window?

Yes. We recorded over a five minutes time widow, but we analyzed one-minute time widow during recovery (1^st^, 3^rd,^ and 5^th^).

L155: over a 1-min time window? From end EX– 1 min to end EX ?

No. We adopted the 30 seconds time window when the volunteers reached 85% of their maximum predicted heart rate.

**# New words in the Material and Methods, page 7, lines 157 and 158**

*…,i.e., ≤ one minute,… … (30 seconds final)…*

L158: please move this paragraph before the description of the analysis you conducted on HR/HRV indices, otherwise it is hard to follow

We adjusted as requested.

L158: this is a very important part for the study outcomes being HRR/HRV recovery influenced by the exercise intensity domain. Please clarify that 85% is the target to be reached in terms of predicted HRmax. Is there any other measurement that can inform about exercise intensity from a metabolic point of view? VO2?

Thank you for the questions. Regarding 85% being the target to be reached over the incremental sub-maximal test, we highlighted on page 7 lines 166 and 167 "and stopped when participants reached 85% of their maximum predicted HR (HR_peak_) by the Tanaka formula [23]." Concerning other measurements from a metabolic point of view as the VO2, unfortunately, we did not measure the maximum oxygen consumption due to our laboratory's lack of a gas analyzer (ergospirometry). Thus, we did not perform the maximum test but a submaximal one (even though the studied population did not present risk factors) for our greater safety.

L164: were participants allowed to run? The average duration of the test is 7.5 min, according to the protocol at 7.5 min the exercise intensity is 9 km/h. Was the 2 initial min enough to reach a steady value of HR before further increasing the treadmill velocity?

This is an excellent question; the answer is yes; the volunteers were allowed to run when they felt like running. However, the volunteers usually started to run at around 7 km/h (3^rd^ minute of the test) since the 2.5% grade remained constant throughout the test.

We rewrite the paragraph.

**# New paragraph in the Material and Methods, page 8, lines 186 - 190**

*The submaximal exercise test started with two minutes of warm-up at a speed of 3.0 km/h and 2.5% grade. The grade remained constant throughout the test and during recovery. After the two minutes of warm-up, the submaximal exercise test protocol started at a speed of 4.0 km/h and 2.5% grade, and the speed was increased by 1.0 km/h every minute until participants reached HR_peak_.*

**# New data’s in the Results, Table 1. Page 10,**

*Speed_peak_ (km/h); BG: 11.2 ± 0.9;NG: 11.0 ± 0.9; ES: 0.2; 0.2 (- 0.3 to 0.8); p: 0.40*

L195: trivial effect instead of no effect

We adjusted as requested.

**# New words in the Material and Methods, pages 9 - 10, lines 220, 222 and 225**

*… trivial effect…*

**Results**

Please state in the tables whether the values refer to mean or median values

Thank you very much for the question. We adjusted as requested

**Discussion**

L253: still not clear what RHR profiles means

We adjusted as requested

**# New phrase in the Discussion, page 13, lines 276 and 277**

*… RHR average values …*

L257: how? The two groups have the same average age..so the predicted HRmax is on average the same, the difference in resting HR directly reflects into a different "estimated" chronotropic reserve. What are the potential issues you may face targeting 85% of the predicted HR max in two groups that differ in their resting HR? are there any? Consider discussing this in the limitations section

Thanks for the statement. Despite the same HR_peak_, the NG showed a significantly reduced chronotropic reserve of 10.7 beats/min compared to BG; following the exercise, the HRR at 3rd and 5th minutes were 4.9 beats/min and 6.4 beats/min lower in NG than BG, respectively.

Thus, the RHR average values can explain the variance of HR during exercise and recovery from 11 to 48% in physically active men, independent of the same HR_peak_ within groups. Therefore, the explication regression linear model applied in our study shows no potential issue considering targeting 85% of the predicted HR_max_. Yet, we do not consider discussing the submaximal stress test as a limitation because we did not measure the maximum oxygen consumption due to our laboratory's lack of a gas analyzer (ergospirometry). Thus, we did not perform the maximum test but a submaximal one (even though the studied population did not present risk factors) for our greater safety.

L257: how did you measure para withdrawal during exercise? That is not clear at all. Is there a difference in HR kinetics? Is there a faster HR response in BG? The difference in reactivity refers to the orthostatic task.

It is an excellent question. We measured parasympathetic withdrawal by the difference between rMSSD_peak_ from rMSSD_sup_. According to Laborde, 2018: "Reactivity represents the change between baseline and a specific event, like completing a task, for example cognitive, emotional, or physical. Reactivity to an event or stress is crucial regarding adaptability, and both lower and higher vagal withdrawal can be facilitative when facing demands". Therefore, as well as the parasympathetic response to an orthostatic test and a submaximal exercise test represents a reactivity process.

Laborde, Sylvain, Emma Mosley, and Alina Mertgen. "Vagal tank theory: the three rs of cardiac vagal control functioning–resting, reactivity, and recovery." Frontiers in neuroscience 12 (2018): 458.

**# New phrase in the Material and Methods, pages 7 and 8, lines 176 - 178**

*and we obtained the parasympathetic withdrawal by subtracting rMSSD_peak_ from rMSSD_sup_ through the absolute (∆_abs_rMSSD_exer_) and the relative (∆_%_rMSSD_exer_) rMSSD variation.*

L258: HRR 1st min should reflect parasympathetic reactivation but this is not different in the two groups. Please elaborate on this. HRR 5th min should reflect para reactivation + sympathetic withdrawal occurring during the post-exercise period, but you are not properly discussing this in the manuscript. HRR30s is also often used as a marker of parasympathetic reactivation. Why did you choose not to present those data?

We are thankful to the Reviewer for this plausible and interesting question. We choose not to present HRR_30s_ because there is no difference between groups. Also, the first minute following the exercise is established as a standard time for clinical evaluation; maybe the first minute of recovery is not the most appropriate post-exercise time for evaluating HRR as usually measured.

We relocate and rewrite the paragraphs and add a new paragraph to the text.

**# New paragraph relocated and rewritten in the Discussion, pages 14 and 15, lines 298 - 313**

*Previous studies with similar exercise protocols to our study verified a positive significant correlation between resting parasympathetic activity with the HRR and parasympathetic activity after the submaximal exercise test [9, 11]. Evrengul et al.[11] observed a positive significant correlation between parasympathetic activity at rest and HRR in the 3rd minute during passive recovery (supine position). Danieli et al. [9] observed a positive correlation between resting parasympathetic activity with HRR at 30s, 1st and 2nd minute during passive recovery (seating position).*

*On the other hand, some authors have shown divergent results considering the relationship between resting parasympathetic activity with HRR and parasympathetic activity after the exercise test [8-16]. One of the pioneers in the field was Javorka et al. [12], who did not observe a significant correlation between resting parasympathetic activity with relative HRR and parasympathetic reactivation in the 1st and 5thminute after submaximal exercise. In the same direction, Molina et al. [14, 15] published studies that did not observe a correlation between absolute and relative HRR at the 1st minute with parasympathetic activity in the supine position and the standing position following maximal treadmill exercise testing in healthy men.*

**# New paragraph in the Discussion, pages 15 and 16, lines 314 - 332**

*Considering the present results, we did not observe differences between groups on the HHR, %HRR, and parasympathetic activity in the first minute of recovery; it is an established standard time. Probably the absence of a significant difference in the parasympathetic activity within the first minute following the submaximal exercise test may be due to the coactivation of both autonomic branches (rapid parasympathetic reactivation with simultaneously sympathetic deactivation) and the effort interruption criterion in 85% of predicted maximum HR, which could impact this comparative analysis (BG vs NG).*

*On the other hand, most studies on HRR focused on the recovery in the first minute and the third minute after exercise [8-13] and there are a few literature reports in which both early and late HRR has been examined [14-16]. Additionally, different implications of exercise physiology can be appreciated with a comprehensive analysis of HRR beyond the first minute. After exercise, HRR in the first 30s to 1st min is predominantly driven by parasympathetic reactivation (early phase with rapid decline), whereas in the subsequent two or more minutes (late phase with slow decay), sympathetic withdrawal in addition to parasympathetic reactivation, sets in as a pivotal determinant of heart rate decline [5]. Therefore, BG compared to NG showed faster absolute and relative HRR in the 3rd and 5th minutes of recovery due to higher parasympathetic activity values in the 3rd and 5th minutes of recovery, as shown in Table 3.*

L265: this part is really similar to the first part of the introduction. Please consider rephrasing

We adjusted as requested

**# New phrase in the Introduction, page 2, lines 54 - 58**

*During an incremental exercise test, heart rate (HR) increases due to parasympathetic withdrawal and sympathetic activation [5]. In contrast, after exercise, the short-term post-exercise HR adaptation, the heart rate recovery (HRR), occurs in response to simultaneous rapid parasympathetic reactivation and progressive sympathetic deactivation [5].*

**# New paragraph in the Discussion, page 14, lines 292 - 297**

*It is well-established that the HR increases (chronotropic reserve) during an exercise test due to parasympathetic withdrawal and sympathetic activation, and following the exercise test, the short-term HR adjustment responds to the rapid parasympathetic recovery and progressive sympathetic withdrawal [5]. Therefore, the coactivation of both autonomic branches, parasympathetic and sympathetic, occurs over several minutes immediately after exercise [5, 34].*

L271-L283: this part is not very comprehensible. Please try to make your point more clear rearranging these three sentences and avoiding repetitions

We adjusted as requested.

**# New paragraph in the Discussion, pages 16 and 17, lines 345 - 357**

*Thus, one possibility is that the lower RHR average values (higher parasympathetic activity) and greater parasympathetic reactivity (reduction) after the postural change (orthostatic stress) before an adaptative functional demand as the exercise may be considered a preliminary marker of better chronotropic and parasympathetic responses during and after exercise.*

*Another aspect to be highlighted in our work is that BG showed higher chronotropic reserve and parasympathetic withdrawal during the submaximal incremental exercise test and faster HRR and parasympathetic reactivation after post-exercise compared to NG. Therefore, our results may add to the literature that RHR average values might be considered a cardiac autonomic "flexibility" index, which helps to understand the relationship between RHR, its adjustment, and cardiovascular health, which is important and potentially toll on individuals' care. From a clinical perspective, the results may be considered important to the individual evaluation of cardiovascular health, but this goes beyond our scope and should be investigated in future studies [38, 39].*

*In addition, our findings may add important information based on a novel approach toward developing an explication regression based on the effect of RHR average values on the HR response during these functional conditions (rest, exercise, and recovery) for a better understanding of these complex interactions.*

L288: for a similar resting HR can HRV analysis add some meaningful information to interpret subject's cardiac autonomic profile and responses to stressful tasks?

This is an excellent question. Yes, HRV can add meaningful information to subjects' cardiac autonomic profiles by considering that two individuals can show the same RHR with distinctive parasympathetic activity (i.e., Parasympathetic saturation).

L351: so why have you found the differences 3 and 5 min post exercise? How does resting HR can influence your calculations? If you try to consider resting HR do these differences disappear?

This is an excellent question. These differences in HRR between groups probably were due to higher parasympathetic activity values in the 3^rd^ and 5^th^ minutes of recovery. And the lower RHR average values (higher parasympathetic activity) and greater parasympathetic withdrawal (reactivity) after the postural change (orthostatic stress) at rest before an adaptative functional demand as the exercise, could mean marker of better chronotropic and parasympathetic responses after exercise.

**# New phrase in the Discussion, page 16, lines 330 - 332**

*Therefore, BG compared to NG showed faster absolute and relative HRR in the 3rd and 5th minutes of recovery due to higher parasympathetic activity values in the 3rd and 5th minutes of recovery, as shown in Table 3.*

BG starts from 82 bpm and NG from 88 bpm, they both go up to 164 then down to 97 bpm and 104 bpm 5 min post-exercise. So after 5 min both groups are around + 15 bpm over their initial HR, and the difference seems to be similar to the difference at rest. If you consider the higher starting point for NG group the difference becomes further smaller. So, are they different? Or not?

This part is significant for the general purpose of the manuscript, and it needs to be better presented and discussed.

We do not know. We did not do the correction by chronotropic reserve because possibly we may remove meaningful variance in outcomes of interest that can be attributed to autonomic and neurophysiological phenomena, according to Geus, 2019.

de Geus, Eco JC, et al. "Should heart rate variability be "corrected" for heart rate? Biological, quantitative, and interpretive considerations." Psychophysiology 56.2 (2019): e13287.

What about adding some graphs?

We are thankful to the Reviewer, but it was a style option for data presentation.

How much of the variance in post-exercise HRR (1st, 3rd, 5th min of rec) or in reactivity to an orthostatic test can be explained by the differences in term of resting HR? what percentage of explained variance is enough to safely say that measuring HR/HRV at rest is helpful instead of including other type of measurements? Please clarify.

This is an excellent question. Thank you! The RHR average values do not explain the variance of difference average between the groups the HHR at 1^st^ minute of recovery. According to described in the article (page 17, lines 358 – 369): "The NG presents on average 8.2 bpm in HR_ort_ and 6.2 bpm in HR_initial_ more than the BG, in which the RHR explains 17% and 22%, respectively, of the variance of this difference average between the groups. NG presents on average 5.5 % in ∆_%_RHR, 10.7 bpm in CR, 4.9 bpm in HRR 3^sd^_min_, 6.4 bpm in HRR 5^th^_min_, 3.1 bpm in %HRR3^sd^_min,_ and 3.9 bpm in %HRR5^th^_min_ lower than the BG, in which RHR explain from 11% to 48% o the variance of this difference average between the groups".

Considering the present results of the percentage explained of HR, we can say that RHR average values open the possibility of a new approach (or analysis), which may add helpful information as a preliminary tool for decision-making in stress management for healthcare professionals bringing essential and complementary information related to individual cardiac autonomic capacity.

**# To the Reviewer 2**

1. The second objective of this study is not clear here; to determine whether RHR could explain the outcome of HR responses during 3 tested conditions. Please rewrite this objective so that it can be explained by your tabulated results.

We agree with the Reviewer's consideration of our statement. It improves our study. And, the second objective of this study was not clear, and therefore, we change it according Shmueli, 2010 which describe the explication regression.

**# New phrase in the Introduction, page 4, lines 97-101 (highlighted)**

*Accordingly, our objectives were: (a) to compare the HR and parasympathetic responses during rest, exercise, and post submaximal exercise in physically active young men with different RHR average values; (b) to develop an explication regression based on the effect of RHR average values on the HR response during rest, exercise, and post submaximal exercise in physically active young men.*

Shmueli, Galit. "To explain or to predict?." Statistical science 25.3 (2010): 289-310.

2. What is your primary and secondary outcomes for this study? It seems that you are putting/testing everything together in this one study with only a small sample size. Make sure that you only focus on your main outcome measures.

Thank you very much for the question. The primary outcome, we observed that BG (RHR < 60 bpm) showed high HR and parasympathetic response (modulation), higher chronotropic reserve, and parasympathetic withdrawal (reactivity) during the submaximal exercise test, and faster HRR and parasympathetic reactivation after the submaximal exercise test compared to NG.

Furthermore, the second outcome RHR explained the variance of HR at rest, exercise, and recovery from 11 to 48% in physically active men.

Regarding the small sample size, this study shows that all differences observed were statistically significant with a power greater than 80%, mitigating the possibility of a type I error.

3. What is the reason for choosing male subjects only? Why do you exclude female subjects?

Thank you very much for the question. At the time of conducting this research, we only had this sample available. Second, according to Laborde, 2017, age, sex, physical fitness, and clinical status are considered important confounding variables influencing the analysis of HRV, and they must be controlled.

LABORDE, Sylvain; MOSLEY, Emma; THAYER, Julian F. Heart rate variability and cardiac vagal tone in psychophysiological research–recommendations for experiment planning, data analysis, and data reporting. Frontiers in psychology, v. 8, p. 213, 2017.

4. How do you calculate your sample size?

Thank you very much for the question. As we wrote in the text (page 8; lines 198 – 199): "The observed power (OP) was calculated by post hoc power analyses using G*Power 3.1.9.7 for Windows software [29]". The *post hoc power analyses* often make sense after a study has already been conducted. In post hoc analyses, 1 – β is computed as a function of *α*, the population effect size parameter, and the sample size(s) used in a study. It thus becomes possible to assess whether a published statistical test had a fair chance of rejecting an incorrect H0. Importantly, post hoc analyses, like a priori analyses, require an H1 effect size specification *for the underlying population*. Post hoc power analyses should not be confused with so-called retrospective power analyses, in which the effect size is estimated from sample data and used to calculate the *observed power*, a sample estimate of the true power

At any alpha level, increased sample sizes always yield greater statistical test power, and a potential problem then turns into excessive power. By "excessive," increasing the sample size implies that smaller and smaller effects will be perceived as statistically significant until, in very large samples, almost every effect is significant. The researcher must always be aware that the sample size can impact the statistical test, making it insensitive (with small samples) or overly sensitive (with very large samples).

The relationships between alpha, sample size, effect size, and power are very complicated, and many guidance references are available. Cohen examines power for most tests of statistical inference and guides acceptable levels of power, suggesting that studies should be designed to achieve alpha levels of at least 0.05 with power levels of 80%. To achieve such levels of power, the three factors – alpha, sample size, and effect size – must be considered simultaneously.

According to Laborde, 2017, in terms of sample sizes, the effect size distribution analysis suggests that to achieve 80% power, samples of 21 participants are required to detect large effect sizes. If another effect size or statistical power level is desired, power analysis can be conducted with the help of the G*Power software.

HAIR, Joseph F. Multivariate data analysis. 2009.

FIELD, Andy; MILES, Jeremy; FIELD, Zoë. Discovering statistics using R. 2012.

LABORDE, Sylvain; MOSLEY, Emma; THAYER, Julian F. Heart rate variability and cardiac vagal tone in psychophysiological research–recommendations for experiment planning, data analysis, and data reporting. Frontiers in psychology, v. 8, p. 213, 2017.

5.Your tabulated results are not clear to the readers (see tables 2 and 3). The information in the table should be divided into the 3 conditions; at rest, during, and after.

Thank you very much for the question. We adjusted as requested.

6. For you regression analysis, how do you decide on the variables selection for analysis during this 3 conditions? Why did you have a mixture of absolute variable and relative variable (%) in your analysis?

Thank you very much for the question. We utilized the theoric and only variables that presented significant statistics between groups because we have only one predictor variable (independent variable). Also, all variables utilized on simple linear regression met the assumptions of normality, linearity of parameters, normality of residuals, independent values, homoscedasticity, and absence of autocorrelation of residuals (Durbin-Watson test), absence of multicollinearity, and absence of outliers. Lastly, we used absolute and relative variables (%) data to reinforce the studied phenomenon.

7. You should be cautious in drawing conclusions because this study only includes young active males. Do not generalise your conclusion with your statement about using the autonomic flexibility index to assess individual CVS health.

Thank you very much for the question. I agreed with the review's comments. To avoid generalizing regarding our result, we describe in the discussion section, sub-section limitation (pages 18 and 19, lines 393 – 401): "*Limitations in our work include the exclusion of older subjects, athletes, and women of any age range, so the findings cannot be extrapolated to these groups of people. Also, these results cannot be extrapolated to men in general because our sample was relatively homogenous, and thus men with different clinical/functional conditions would present different responses than those observed in the present study. Additionally, our findings cannot be extrapolated for other workload and duration exercises or protocols like a cycle or arm ergometry, where the individuals sit during rest, exercise and recovery. Also, the participants performed an active recovery after exercise in the orthostatic position, which limits the comparison to similar conditions*".

Conclusion

Lastly, low RHR average values (< 60 bpm) showed to be a cardiac autonomic "flexibility" index for assessing individual cardiovascular health in young, physically active men.

Therefore, these finds **suggest that RHR** may be used as a preliminary tool for decision making, considering its potential helpful information related to individuals' cardiac autonomic capacity without the expense of clinical exercise tests to infer the parasympathetic activity (sympathovagal balance) response in the heart.
