# Supplementary material for: Can resting heart rate explain the heart rate and parasympathetic responses during rest, exercise, and recovery?
Source: PLoS One. 2022 Dec 7;17(12):e0277848. doi: 10.1371/journal.pone.0277848 (PMC9728889; doi:10.1371/journal.pone.0277848)
Supplement: Attachment — Submitted filename: responses_to_reviewers_plos.docx [file pone.0277848.s004.docx]

Responses to the reviewers

2022/10/14

Manuscript title: Can resting heart rate explain the heart rate and parasympathetic responses during rest, exercise, and recovery?

Manuscript ID: PONE-D-22-17046R1.

Authors: Giliard Lago Garcia, Luiz Guilherme Grossi Porto, Carlos Janssen Gomes da Cruz and Guilherme Eckhardt Molina

We thank the Editor-in-Chief of PLOS ONE for the opportunity to resubmit our paper. We are very grateful to the reviewers for their comments and suggestions, which improved our manuscript. As requested, we have highlighted edits to facilitate further review. We believe the responses and additions satisfactorily address the reviewers' comments and suggestions and hope you will agree. The edits have clarified several points and have resulted in an improved manuscript, which we hope is now ready for final acceptance.

**# To Journal Requirements:**

Please review your reference list to ensure that it is complete and correct. If you have cited papers that have been retracted, please include the rationale for doing so in the manuscript text or remove these references and replace them with relevant current references. Any changes to the reference list should be mentioned in the rebuttal letter that accompanies your revised manuscript. If you need to cite a retracted article, indicate the article’s retracted status in the References list and also include a citation and full reference for the retraction notice.

All references are correct.

**# To the Reviewer 1**

**Abstract:**

L28: please delete “average”, that is implicit (throughout the manuscript as well)

We adjusted as requested.

L31: delete “and HR” that is implicit too as you measured the RR intervals

We adjusted as requested.

**Introduction:**

L85: please re-examine this sentence: first delete “average”, then it is not clear how the HR can be both the independent and the dependent variable at the same time.

You stated that it is important to know the effect of resting HR on HR at rest, which does not make any sense. You may want to test the hypothesis that different values of HR at rest are associated with different values of HRV, and different exercise and post-exercise responses etc.

We thank the Reviewer's question. To avoid duality (misinterpretation) considering RHR and HR at rest, we adjusted the sentence throughout the manuscript.

**# New phase in the Introduction, page 2, line 53 (highlighted)**

*…, evaluated in the supine position,…*

**# New phase in the Introduction, page 4, lines 84 - 87 (highlighted)**

*To the best of our knowledge, no studies have shown information on the effect of different RHR values, evaluated in the supine position, on HR dynamic and parasympathetic activity at rest, during the exercise test, and recovery.*

Also, the RHR evaluated in the supine position is the only independent variable as we highlighted in the Material and Methods section, page 6, lines134 - 138: *“To test our hypothesis, the subjects were allocated into two groups based on the supine RHR values. Those who presented bradycardia at rest (RHR < 60 bpm) were grouped in the bradycardia group (BG n = 20), and those who presented RHR values within the normal range (HR ≥ 60 bpm < 100 bpm) were allocated to the normal HR group (NG n = 20).”.* Accordingly, all other measures of HR **dynamics** and parasympathetic activity assessed at rest, during the exercise test, and during recovery are **dependent variables**.

Thus, to the best of our knowledge, no studies have shown information on the effect of different RHR (independent variable) values on HR dynamics (dependent variable) and parasympathetic activity (dependent variable) at rest, during the exercise test, and recovery.

**# New phase in the Introduction, page 4, lines 95 - 97 (highlighted)**

*Therefore, we hypothesize that physically active young men with different RHR values in the supine position can show different HR dynamics and parasympathetic activity at rest, during, and after a submaximal exercise test.*

L94: please consider making your hypothesis clearer. In which direction do you expect these relationships to go? e.g. A lower RHR = a faster recovery or a slower recovery? Obviously based on the literature you have already mentioned

We appreciate the Reviewer's question. However, we would like to highlight that this paper is not a correlational study but a comparative study. Thus, as we described in the Material and Methods section, page 10, line 227: “*The two-tailed level of statistical significance was set at a p ≤ 0.05*.” Therefore, our **null hypothesis** was that physically active young men with different RHR values in the supine position show **the same** HR dynamic and parasympathetic activity at rest, during, and after a submaximal exercise test, and our **alternative hypothesis** was that physically active young men with different RHR values in the supine position can **show different HR dynamics** and parasympathetic activity at rest, during, and after a submaximal exercise test.

**# New phase in the Introduction, page 4, lines 95 - 97 (highlighted)**

*Therefore, we hypothesize that physically active young men with different RHR values in the supine position can show different HR dynamics and parasympathetic activity at rest, during, and after a submaximal exercise test.*

**Methods:**

How did you perform the regression analysis? Was the predictor variable RHR considered as a continuous variable? Or you coded the two groups (e.g. BG = 0, NG=1)? please consider the first option as the best way to perform a linear regression analysis. What are the advantages of using the mean HR difference between the two groups instead of the raw values of RHR? Please clarify.

We are thankful to the Reviewer for this plausible and interesting question. We did not consider the predictor variable RHR (independent variable) as a continuous variable. We considered the predictor variable RHR as a dummy variable (BG = 0, NG=1) because we aim to develop an explication regression based on the effect of RHR values on the HR dynamics response during rest, exercise, and post-submaximal exercise in physically active young men, according to Shmueli, 2010 which describe the explication regression.

The predictive models tend to have higher predictive accuracy than explanatory statistical models; they can indicate the potential level of predictability. A very low predictability level can lead to the development of new measures, newly collected data, and new empirical approaches. An explanatory model close to the predictive benchmark may suggest that our understanding of that phenomenon can only be increased marginally.

On the other hand, the explanatory model advantage is close to the predictive benchmark. Thus, the explanatory model would imply substantial practical and theoretical gains in further scientific development. Hence, explanatory modeling is commonly used for theory building and testing.

Shmueli, Galit. "To explain or to predict?." Statistical science 25.3 (2010): 289-310.

**Results:**

You may want to add the ES descriptor (e.g. small/trivial etc..) in the tables, that will help the reader interpret your results. I also suggest mentioning (and discussing) the size of the effects in the following paragraphs to help the reader throughout the long discussion.

Thank you very much for the question. We add the effect size descriptor in the tables as requested. We added the effect size descriptor as requested above by the reviewer.

**Discussion:**

I appreciate the effort done by the authors in clearly presenting their results, statistical power, etc. but how these trivial-to-small differences between the two groups in terms of exercise/post-exercise recovery responses and such small portions of the variance explained by the regression model can be translated into something helpful? How does this help clinical practice?

This is an excellent question. Thank you! According to described in the article (page 13, lines 270 – 275): " *The NG presents on average 8.2 bpm in HR_ort_ and 6.2 bpm in HR_initial_ more than the BG, in which the* ***RHR explains*** *17% and 22%, respectively, of* ***the variance of this difference average between the groups****. NG presents on average 5.5 % in ∆%RHR, 10.7 bpm in CR, 4.9 bpm in HRR 3^rd^_min_, 6.4 bpm in HRR 5^th^_min_, 3.1 bpm in %HRR3^rd^_min_, and 3.9 bpm in %HRR5^th^_min_ lower than the BG, in which RHR explain from 11% to 48% o the* ***variance of this difference average between the groups****."*.

Considering the present results of the percentage explained variance of difference average between the groups, we can say that RHR opens the possibility of a new approach (or analysis), which may add helpful information as a preliminary tool for decision-making in stress management for healthcare professionals bringing essential and complementary information related to individual cardiac autonomic capacity.

**# New paragraph in the Discussion, page 17, lines 352 - 359 (highlighted)**

*Thus, the novelty of the present study is that these results may open a new possibility to the RHR (supine) analysis as a preliminary tool for decision-making, considering its potential helpful information related to the individual’s cardiac autonomic “flexibility” without the expense of clinical exercise tests to infer the parasympathetic activity on the heart in different functional conditions (rest, exercise, and recovery). From a clinical perspective, due to the presence of a small to large effect size, these results may be considered important to assess cardiovascular health, but this goes beyond our scope, and new studies should be done to confirm this hypothesis with the clinical population.*

I also wonder whether correlation analysis, rather than group analysis, could somehow help explain how resting, exercise and post-exercise responses are related. Why did you choose this kind of approach?

Thank you very much for the question. First, the approach through correlation analysis can be a bit simplistic because it only presents the results of the direction and strength of the correlation. Thus, the correlation between the variables X and Y can arise either from X affecting Y or from Y affecting X. Sometimes X and Y can be cause and effect at the same time.

However, the simple linear regression analysis is more robust and requires that several assumptions are met to use it. Therefore, all variables utilized on simple linear regression were decided by the theoric, variables correlated with the independent variable, and only variables that presented significant statistics between groups because we have only one predictor variable (independent variable).

All variables met the assumptions of normality, linearity of parameters, normality of residuals, independent values, homoscedasticity, absence of autocorrelation of residuals (Durbin-Watson test), absence of multicollinearity, and absence of outliers (Godfrey, 1985; Bewick, 2003).

Different from correlation analysis, simple linear regression not only test for relationships between variables but also quantifies their direction and strength. Thus, simple linear regression presented more robust measures than correlation analysis, such as the coefficient of determination (R^2^) and the regression coefficient (slope) (Hair, 2009).

Which, the coefficient of determination then represents the adjustment between the dependent variable (Y) and the independent predictor (X) variables that designate the effect. In other words, the coefficient of determination is the proportion of the variance of Y that can be predicted or explained based on its linear relationship with X. The stronger the coefficient of determination, the stronger the prediction or explanation of the association between the dependent variable Y and the independent variable X in linear terms. The minimum theoretical value of the coefficient of determination is 0% and the maximum is 100%, although the maximum is not expected in the health sciences (Espirito Santo, 2018). Also, remember that the coefficient of determination value is simply the squared correlation between the actual and predicted values. (Hair,2009).

The regression coefficient (slope) describes the average (expected) change in the dependent variable for each unit change in the independent variable. If the regression coefficient is perceived to be statistically significant (ie, the coefficient is significantly different from zero), the value of the regression coefficient indicates the extent to which the independent variable associates with the dependent one. (Hair, 2009)

Wherefore, according to described in the article (page 17, lines 360 – 363): “In addition, our findings may add important information based on a novel approach toward developing **an explication regression** based on the effect of RHR values on the HR response during these functional conditions (rest, exercise, and recovery) for a better understanding of these complex interactions”.

Bewick, Viv, Liz Cheek, and Jonathan Ball. "Statistics review 7: Correlation and regression." Critical care 7.6 (2003): 1-9.

Godfrey, Katherine. "Simple linear regression in medical research." New England Journal of Medicine 313.26 (1985): 1629-1636.

Hair, Joseph F. "Multivariate data analysis." (2009).

Espirito Santo, Helena, and Fernanda Daniel. "Calcular e apresentar tamanhos do efeito em trabalhos científicos (3): Guia para reportar os tamanhos do efeito para análises de regressão e ANOVAs [Calculating and reporting effect sizes on scientific papers (3): Guide to report regression models and ANOVA effect sizes]." Revista Portuguesa de Investigação Comportamental e Social 4.1 (2018): 43-60.

These two points need to be presented and discussed in the manuscript.

**# New paragraph in the Conclusion, page 20, lines 422 - 426 (highlighted)**

*Therefore, we suggest that RHR (supine) analysis as a preliminary tool for decision-making, considering its potential helpful information related to the individual’s cardiac autonomic “flexibility” without the expense of clinical exercise tests to infer the parasympathetic activity on the heart in different functional conditions (rest, exercise, and recovery).*
